# Access to Vaccines Among Asylum Seekers, Refugees, and Undocumented Migrants Across the Migratory Cycle in the European Union, European Economic Area, Switzerland and the United Kingdom: A Scoping Review

**DOI:** 10.3390/vaccines14060551

**Published:** 2026-06-22

**Authors:** Saleh Aljadeeah, Anil Babu Payedimarri, Carine Dochez, Karina Kielmann, Veronika J. Wirtz, Sally Hargreaves, Raffaella Ravinetto

**Affiliations:** 1 Department of Public Health, Institute of Tropical Medicine, 2000 Antwerp, Belgium; cdochez@itg.be (C.D.); rravinetto@itg.be (R.R.); 2 Division of Hematology, Department of Translational Medicine, AOU Maggiore della Carità di Novara, University of Eastern Piedmont, 28100 Novara, Italy; anil.payedimarri@uniupo.it; 3 Research Training Innovation Infrastructure, Research and Innovation Department (DAIRI), Azienda Ospedaliero-Universitaria SS. Antonio E Biagio E Cesare Arrigo, Via Venezia, 16, 15121 Alessandria, Italy; 4 Institute for Global Health and Development, Queen Margaret University, Edinburgh EH21 6UU, UK; kkielmann@qmu.ac.uk; 5 Department of Global Health, Boston University School of Public Health, Boston, MA 02118, USA; vwirtz@bu.edu; 6 Migrant Health Research Group, Institute for Infection and Immunity, City St George’s, University of London, London SW17 0RE, UK; shargrea@citystgeorges.ac.uk; 7 School of Public Health, University of The Western Cape, Cape Town 7535, South Africa

**Keywords:** vaccine, access, asylum seekers, refugees, undocumented migrants, Europe

## Abstract

**Introduction**: Inequities in access to medicines persist for asylum seekers, refugees, and undocumented migrants in Europe. For vaccines, access gaps not only exist for these groups in childhood routine immunization, but also for life-course and catch-up vaccinations. As part of a broader project examining access to medicines and vaccines for migrants across all stages of the migration cycle, this scoping review synthesizes evidence on the determinants of access to vaccines. **Methods**: We conducted a scoping review across PubMed, Cumulative Index to Nursing and Allied Health Literature (CINAHL), Cochrane Database of Systematic Reviews, Scopus, and grey literature sources, covering the period 2000–2024. Sources were eligible if they addressed access to vaccines among migrants. We examined access to vaccines along the life course, and across phases of the migratory cycle, including departure, transit, reception and settlement, and return or deportation. **Results**: A total of 47 research studies and grey literature reports were included. Most studies focused on migrants in reception and settlement (destination) settings, with only twelve sources addressing other phases of the migratory cycle. Across European countries, migrants were frequently reported to have lower uptake of routine vaccines (e.g., measles–mumps–rubella (MMR), polio, diphtheria–tetanus–pertussis (DTP), and human papillomavirus (HPV)) and COVID-19 vaccines than host populations. The most frequently reported barriers were related to migrants’ legal status, administrative requirements, and lack of documentation, alongside poor affordability of vaccination, limited awareness of their rights, and mistrust in the health system. **Conclusions**: Health systems need to adopt innovative approaches to expand vaccine access for migrant populations. Further, protecting confidentiality is essential for building trust and reducing ethical and legal risks. Flexible and coordinated vaccination strategies are required to address migrants’ mobility across the different migration stages and settings. Our findings appeal for sustained improvements in access to vaccines among migrants in Europe, contingent on strong policy commitments to equity, data protection, and the adoption of life-course and catch-up vaccination strategies.

## 1. Introduction

Vaccination is one of the most effective public health interventions in history, preventing millions of deaths and contributing to the control and elimination of numerous infectious diseases [1]. Yet, universal access to vaccines is not guaranteed. Vaccination coverage remains an indicator of inequity: those who miss out on vaccines often face broader exclusion from essential health services [1,2]. Groups most affected by inequity include refugees, asylum seekers, and undocumented migrants [3]. Despite regional and global statements of commitment to “leave no one behind” [1,2], these populations have lower vaccination coverage and higher burdens of vaccine-preventable diseases (VPDs) compared with host populations in the countries of transit or arrival in Europe [1,4].

The World Health Organization (WHO) European Region has achieved significant progress in expanding immunization coverage, yet important gaps still affect migrants [5,6], as seen during the COVID-19 pandemic. In the United Kingdom (UK), a cohort study of more than 465,000 migrants showed that they had slower uptake of the first dose of COVID-19 vaccines across all age groups versus the general population [3,7]. Similar patterns were reported in other European countries, where barriers to vaccination exacerbated existing vulnerabilities, resulting in unequal protection from COVID-19 and other infectious diseases [4,8]. The European Vaccine Action Plan 2015–2020 and the subsequent EU Vaccine Strategy (2020) reaffirmed vaccination as a key public health priority, aiming for ≥95% coverage for measles–mumps–rubella (MMR) vaccination, sustained control of hepatitis B, and sustained polio-free status [9,10]. However, achievement of these goals is hindered when specific groups are left behind; access to vaccination among migrants does lag behind the general population, hindered by administrative and financial barriers, and fragmented entitlements [9,11].

Mobility across and within European borders intersects with administrative systems that can either enable or restrict access to vaccines. In many European countries, individuals are required to provide identity or residency documents, or health insurance cards, as a prerequisite for vaccination. These rules may deter or prevent migrants from seeking vaccination services [8], or indeed induce fear of being reported, even in countries where documentation is not legally required. Migrants who have experienced situations of exclusion, racism, xenophobia, or other forms of discrimination in prior interactions with health services or state authorities may develop distrust towards institutions, and, consequently, avoid seeking care [8].

The literature encompasses a wide range of study designs and grey literature sources in this field, making a scoping review particularly appropriate for mapping the breadth of available evidence, identifying knowledge gaps, and exploring how barriers and determinants of vaccine access vary across migration stages. Scoping reviews are designed to systematically map the available evidence on a particular topic, concept, or field of inquiry, drawing on diverse evidence sources to provide an overview of existing knowledge. They are particularly useful for clarifying concepts, identifying key themes and determinants, and examining methodological characteristics and gaps within the literature [12]. Several studies have investigated vaccination among migrants in Europe, but they have typically focused on specific diseases (e.g., COVID-19) and national contexts. Some reviews have examined the determinants of vaccine uptake among migrants in Europe, the drivers of HPV vaccine uptake, and the vaccination delivery models for migrants and refugees; however, their focus was on destination settings only [4,13,14]. They did not examine access to vaccines across the other phases of the migratory cycle, nor have they systematically considered asylum seekers, refugees, and undocumented migrants within a European context. As a result, evidence remains fragmented across settings and migration stages [15].

The current review is one component of a broader project to review the evidence on access to medicines among asylum seekers, refugees, and undocumented migrants in Europe. Findings specific to medicines have been published separately [16]. Vaccines emerged as a distinct component, with a more complex set of barriers to, and determinants of, access, necessitating separate analysis.

For the scope of this work, we refer to ‘Europe’ as countries belonging to the European Union (EU) and/or the European Economic Area (EEA), in addition to Switzerland and the UK. Further, we use the term ‘migrants’ to refer collectively to asylum seekers, refugees, and undocumented migrants. An asylum seeker is a person who has applied for international protection and whose application is still awaiting a final decision by the authorities of the country where the claim was submitted [17]. A refugee is defined under the 1951 Geneva Refugee Convention as a person who is unable or unwilling to return to their country of origin because of a well-founded fear of persecution on grounds such as race, religion, nationality, membership of a particular social group, or political opinion [18]. Undocumented migrants are individuals who do not meet the legal requirements established by the country of destination for entry, residence, or engagement in economic activity [19].

## 2. Materials and Methods

### 2.1. Study Design

In line with Arksey and O’Malley’s framework for scoping reviews [20], refined by Levac et al. [21], we applied an iterative five-stage process, and reported our findings in accordance with the PRISMA-ScR (Preferred Reporting Items for Systematic Reviews and Meta-Analyses extension for Scoping Reviews) checklist [22] (Supplementary File S1). We published the overall detailed study protocol [23] and registered it with the Open Science Framework [23].

### 2.2. Aim

Our overarching aim was to assess key determinants of access to essential medicines and vaccines for migrants along the migration cycle. We set two specific questions for the broader scoping review which covered both medicines and vaccines, namely: (a) What are the patterns of access to essential medicines and vaccines for migrants, including differences across asylum seekers, refugees and undocumented migrants; acute and chronic conditions; preventive and curative interventions; and different diseases? (b) Which barriers limit migrants’ access to essential medicines and vaccines at different stages of the migratory cycle, including how these barriers hamper access to curative care, to preventive care, and if any migration contexts are particularly under-represented? Here, we report on how access to vaccines, specifically, is shaped by determinants acting at different stages of the migration cycle among migrants residing in or aiming to reach Europe.

### 2.3. Database Searches

The search strategy was developed around three key concepts: (1) population (asylum seekers, refugees, and undocumented or irregular migrants), (2) concept (access to medicines and vaccines and related barriers and facilitators), and (3) context (stages of the migration cycle, including origin, transit, destination, and deportation or return) [13,17]. Searches were conducted in PubMed, CINAHL, the Cochrane Database of Systematic Reviews, Web of Science and Scopus. In addition, grey literature sources were searched, including Google Scholar, Open Grey, websites of non-governmental organizations, and websites of relevant international agencies such as the WHO, United Nations High Commissioner for Refugees (UNHCR), United Nations Children’s Fund (UNICEF), Médecins Sans Frontières (MSF), Médecins du Monde (MdM), and the International Committee of the Red Cross. Search terms combined controlled vocabulary and free-text keywords related to migrant populations, medicines and vaccines, access and barriers to access, and migration cycle stages using Boolean operators.

The detailed search terms are presented in Supplementary File S2. The initial search, conducted for the broader scoping review, covered the period 1 January 2000–20 December 2022. For the present vaccine-focused analysis, we expanded the search to include records published from 1 January 2023 to 31 December 2024, applying the same search strategy and inclusion criteria. All results were imported into Rayyan reference manager [24].

### 2.4. Selection of Research and Grey Literature

**Figure 1:**
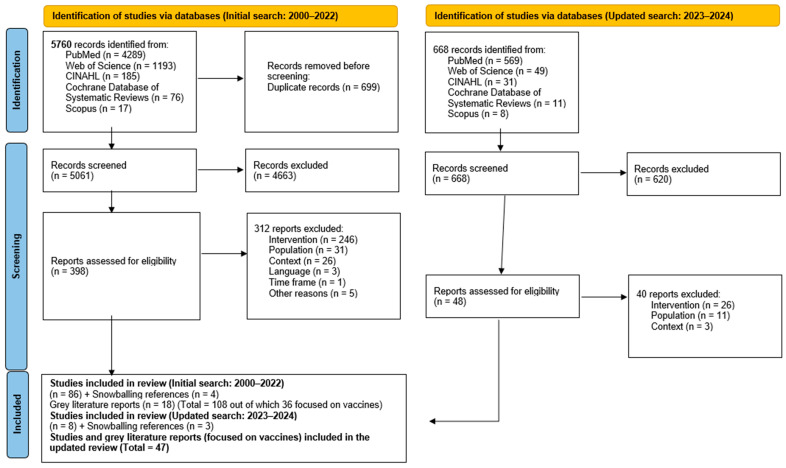
PRISMA flowchart of study selection. From: Page MJ, McKenzie JE, Bossuyt PM, Boutron I, Hoffmann TC, Mulrow CD et al. The PRISMA 2020 statement: an updated guideline for reporting systematic reviews. BMJ 2021–372:n71. doi: 10.1136/bmj.n71 [26]. For more information, visit: http://www.prisma-statement.org/ (accessed on 17 June 2026).

Screening followed a two-phase process [16]. In the first phase, titles and abstracts were screened against predefined eligibility criteria (Supplementary File S3). Eligible sources included studies and reports published in English between 1 January 2000 and 31 December 2024 that addressed access to vaccines among asylum seekers, refugees, or undocumented migrants. Studies focusing on other migrant populations or on healthcare access without specific reference to medicines or vaccines were excluded. In the second phase, full texts of potentially eligible publications were independently assessed by two reviewers (SA and ABP), with discrepancies resolved through discussion and arbitration by a third reviewer (RR) until consensus. In addition, the reference lists of included publications were screened to identify further relevant studies and reports that had not been retrieved through the database and grey literature searches. The number of records identified, screened, excluded, and included at each stage of the selection process is presented in the PRISMA flow diagram (Figure 1) [25].

### 2.5. Extracting and Charting Data

A structured data-extraction matrix was created, based on the research questions. The matrix was refined through an iterative process, whereby two reviewers initially entered data for five eligible studies to assess the extent to which the matrix captured relevant data before moving to full data extraction. The extraction form included bibliometric information (e.g., authors, year of publication, country, study objectives, study design, and characteristics of the study population), as well as analytical variables relevant to the review questions, including migration cycle phase, vaccine type, patterns of vaccine uptake, barriers and facilitators of access, interventions to improve vaccine access and uptake, and other key findings.

### 2.6. Collating, Summarizing, and Reporting Results

Given the heterogeneity of the included evidence, comprising quantitative, qualitative, mixed-methods, review studies, and grey literature reports, extracted data were synthesized using a descriptive and narrative approach. Descriptive bibliometric characteristics (e.g., publication year, country, study population, and study design) were summarized quantitatively, while narrative findings relating to vaccine access and uptake were grouped according to the identification of common themes, relating to determinants, barriers, facilitators, interventions, and policy recommendations. Findings are reported according to the PRISMA Extension for Scoping Reviews guidelines. We interpret these findings and their implications for future research and practice and highlight gaps in the academic and grey literature that warrant attention in future research.

### 2.7. Defining Concepts of Migration Cycle and Vaccine Uptake

The migration cycle stages were defined according to the WHO framework “Continuum of care during the migration cycle” [27], which builds on the conceptual models developed by Zimmerman et al. [28] and Thomas [29]. This framework recognizes the dynamic nature of migration, encompassing the country of origin, departure, transit, destination, and (when applicable) return or deportation.

To conceptualize determinants of vaccine uptake, we employed the 5As framework (Access, Affordability, Awareness, Acceptance, and Activation) identifying five interrelated components [30]:

- Access: The ability to reach or be reached by vaccination services.
- Affordability: Economic and opportunity costs of vaccination relative to willingness and ability to pay.
- Awareness: Knowledge and understanding of vaccine recommendations and availability.
- Acceptance: Attitudes and trust regarding vaccines and systems delivering them.
- Activation: Interventions or prompts that encourage individuals to get vaccinated.

We adopted a life-course perspective on vaccination [30], distinguishing between routine childhood immunization and life-course or catch-up vaccination, and reflecting differences in delivery models, policy frameworks, and barriers to access across age groups.

## 3. Results

Our initial search (2000–2022) identified 5760 records, of which 36 studies focusing on access to vaccines were included after screening. The additional search (2023–2024) yielded the inclusion of an additional eight studies and reports, and reference screening yielded three additional studies. Overall, 47 studies and grey literature reports were included in the review (Figure 1).

### 3.1. Characteristics of Included Sources

**Figure 2:**
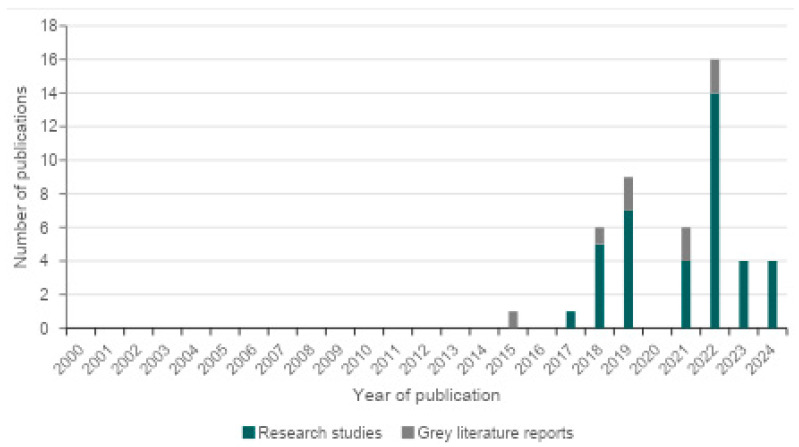
Number of publications (2000–2024) included in the current analysis.

Out of the 47 included sources, 39 were research studies, and eight were grey literature reports. The research studies employed different methods, including quantitative methods (*n* = 16), reviews (*n* = 12), and qualitative methods (*n* = 9) (Supplementary File S4). All were published after 2015 (Figure 2).

### 3.2. Geographic Scope and Migrant Groups

Out of 47 sources, 21 (45%) covered more than one European country, while the remaining focused on a single country. The highest number of studies were conducted in the UK (seven), followed by Italy (five) and Germany (three). Various groups of migrants (asylum seekers, refugees, undocumented migrants) were included in 31 sources, while nine focused exclusively on refugees, four on undocumented migrants, and two on asylum seekers (Supplementary File S4). Fourteen of the included sources were primary empirical studies, contributing individual-level data on 36,745 migrants. Where nationality was reported, the largest groups originated from Syria, Iraq, and Afghanistan, with additional representation from Sudan, Guinea, Somalia, Eritrea, the Democratic Republic of Congo, and Angola.

### 3.3. Migration Cycle Phase

Out of 47 sources, 26 (55%) examined access to/uptake of vaccines in the reception and host country. Two studies from Greece addressed the transit phase [31,32]; one focused on the departure phase [33]; and nine covered multiple phases of the migration cycle [4,9,34,35,36,37,38,39,40]. The remaining nine sources consisted of reports addressing vaccine access among migrants in Europe without a specific focus on a particular stage of the migration cycle and were therefore not assigned to an individual migration phase [10,41,42,43,44,45,46,47,48]. High mobility during transit and staying in informal settlements were factors associated with disrupted follow-up and incomplete multi-dose vaccination, including for COVID-19 [31,32].

### 3.4. Vaccination Patterns and Rates

Out of 47 sources, 32 (68%) addressed access to multiple vaccines. The most commonly investigated vaccines were measles–mumps–rubella (MMR) [13,31,33,38,42,49,50,51]; polio [10,13,33,39,42,43,44,51]; hepatitis B [13,31,38,42,43,44,45,49,51,52]; and HPV [37,48,50,52]. Fourteen sources focused on a single vaccine; 13 on COVID-19 vaccines [4,7,14,40,46,47,53,54,55,56,57,58,59]; and one on the HPV vaccine [60] (Supplementary File S4). One study reported that some migrants were unable to name previously received vaccines [61].

Migrants were frequently reported to have lower vaccination uptake and completion compared with host populations, both for routine vaccines, including measles, polio, diphtheria–tetanus–pertussis (DTP), tetanus, and HPV, and for the COVID-19 vaccine [4,29,33,44,45,48,52,53,54,57,58]. Such gaps resulted in outbreaks of measles, polio, diphtheria, and hepatitis A in migrant camps and settlements, predominantly affecting adolescents and adults [36,62,63].

Although sources often focused on children and adolescents, evidence indicates that vaccination coverage in adults is also low. In the UK, vaccination rates among adult refugees were poorly aligned with national immunization schedules, particularly for DTP [33]; older migrants (≥65) were more likely to be overdue for their second and third COVID-19 vaccine doses versus the host population [7]. Studies from Greece and Italy reported particularly low COVID-19 vaccine uptake in transit settings, with significant delays versus host populations [3,34,57]. Overall, evidence suggests that adult migrants are under-vaccinated, highlighting important gaps in life-course and catch-up immunization that warrant further investigation. Many adults remain incompletely vaccinated or even excluded from vaccination policies, despite WHO and ECDC recommendations to promote “better late than never” strategies as effective and cost-efficient [41,64,65]. One report noted mismatches between vaccine demand and supply, underscoring the risk of wasted doses and missed vaccination opportunities [55].

### 3.5. Barriers and Determinants of Vaccine Uptake

Access was the most frequently reported determinant of vaccine uptake (Table 1). Legal barriers and registration requirements often shaped migrants’ ability to access vaccines [4,10,31,41,44,45,46,59]. Affordability of vaccines or migrants’ reported concerns about costs were a further reported barrier, including direct costs, e.g., migrants’ reported uncertainty regarding whether a vaccination would be provided for free, and indirect costs, e.g., transport to the vaccination centre and time away/income lost from work [10,47,48,51,53,64]. Further, access was reported to be hindered by limited awareness of entitlements, and knowledge of where to access vaccination [46,53,55,63]. Vaccine-related hesitancy and (mis)trust were frequently reported as barriers, particularly for COVID-19 vaccines. This aspect also included mistrust towards the (health) system, fear that personal and medical data might be shared with immigration authorities, concerns about vaccines safety, and misinformation [40,47,53,55,58,65]. Lower uptake of the HPV vaccine among adolescent refugee girls was also linked to poor acceptability [60]. Broader cultural influences (e.g., misconceptions on vaccines/vaccination, low perceived need, or religious beliefs) also reportedly influenced vaccine acceptance and hesitancy [10,42]. Activation was less frequently reported compared to other determinants.

### 3.6. Other Barriers to Vaccine Uptake and Access

At the individual or family level, language barriers were reported in 18 studies [3,4,7,13,37,40,41,49,54,56,60,64,65,66,67,68], and discrimination, xenophobia, and stigma in six sources [4,7,24,47,53,62]. Nine sources reported concerns that personal or medical data might be shared with immigration authorities, resulting in loss of privacy and protection, and consequent risk of deportation or arrest [13,39,46,51,53,54,56,64,65]. Two studies described vaccination fatigue or fear of hyperimmunization [30,63]; two the difficulties of navigating health systems [48,58,60]; and two, vaccine shortages and lack of equity in the distribution of available stocks [53,66].

Several sources highlighted systemic barriers. A recurring theme was the absence of standardized vaccination documentation and of efficient mechanisms for sharing medical and vaccination data across health institutions and health systems along the migration cycle [33,35,47,49,59,63,69]. Reliance on verbal histories was frequently reported as unreliable [69], possibly causing unnecessary revaccination [66].

Some studies underscored the role of legal and administrative status [52,55,70]. Undocumented parents faced structural barriers, e.g., being questioned about their legal status when seeking healthcare for their children; conversely, trust in frontline nurses at child health centres facilitated uptake [70]. These findings confirm that precarity in legal status can delay or disrupt timely vaccination. High population mobility was linked to incomplete vaccination series and lower coverage for second doses [31]. Migrants in transit or in informal settlements were less likely to complete multi-dose vaccination schedules, including COVID-19 vaccination [3]. Frequent relocations between camps or accommodation sites disrupted follow-up and continuity of care [65].

### 3.7. Interventions to Improve Access and Uptake

Engaging migrants in primary care and preventive services remains challenging, particularly for undocumented migrants. The findings of the International Organization for Migration (IOM) EQUI HEALTH project underlined that discrepancies in entitlements to statutory health services across EU/EEA countries exacerbate inequities [13]. Proposed corrective interventions often emphasized community-centred and culturally sensitive strategies, including engagement of trusted leaders, tailored communication, and multilingual information, as measures to address mistrust and misinformation [4,10,14,34,35,37,41,44,48,53,55,58,62,64,65,66,70]. Flexible, low-threshold delivery models such as outreach, out-of-hours clinics, pharmacy-based vaccination, and integration with primary care or arrival health checks were also proposed to reduce structural barriers [4,13,33,36,38,39,48,49,50,63,64,66].

Other proposed strategies include catch-up vaccination initiatives for adults and adolescents [4,33,38,41,48,64,65]; the use of digital tools, e.g., electronic vaccination cards and cross-border registries, to enhance continuity of access [34,35,38,46,48,62]; and the explicit inclusion of all migrant groups (children, adolescents, and adults, including undocumented migrants) in national immunization policies and catch-up programs [9,10,14,31,38,40,41,42,45,51,53,54,63,64,68]. Equitable access to vaccines such as polio and COVID-19 was highlighted as essential to prevent outbreaks, and to ensure genuinely voluntary, confidential, and non-stigmatizing approaches [10,42,44,45]. Interventions are seen as most effective when combining trust-building, low-barrier delivery, and strong measures for the protection of personal and medical data.

Many authors urged that vaccination should be free of charge, protected by medical confidentiality, and supported by simplified administrative procedures [10,40,42,48,53,54,63]. The strengthening of data and surveillance systems appears critical. There is a clear need for harmonized immunization registries, systematic recording of vaccination histories, and improved data sharing to ensure continuity of care [9,35,36,39,40,41,47,49]; however, these vaccination data should not be collected at the expense of data protection [3]. Furthermore, study authors often appealed for sustained financing and training the health workforce, and for participatory approaches that engage migrant communities in service design [7,10,34,40,41,46,47,48,64].

## 4. Discussion

This review identified multiple determinants shaping access to vaccines among migrants in Europe, and the persistent inequities in vaccination uptake among migrant versus host populations. Inequities seem ubiquitous across all vaccine types. Barriers extend beyond individual-level determinants to encompass systemic and structural challenges: fragmented data systems, lack of standardized vaccination documentation, gaps in catch-up vaccination strategies (particularly for adults), and lack of community-centred interventions, such as culturally tailored communication and flexible delivery models. Legal precarity and administrative requirements further delayed or disrupted access, particularly for undocumented and newly arrived migrants.

The evidence on migrants’ access to vaccination (as well as to medicines [14]) has expanded considerably over the past decade, with a marked increase in publications between 2017 and 2022. This likely reflects heightened attention following the influx of asylum seekers to Europe since 2014, and growing recognition of immunization gaps in displaced populations [14]. The COVID-19 pandemic has triggered more attention, possibly due to broader concerns for European health security [71].

Although many sources focused on childhood and adolescent vaccination, most asylum seekers and refugees in Europe are adults. In 2024, minors accounted for approximately one-quarter of asylum applicants in EU countries, while about one-half were aged 18–34, and the remaining one-quarter were older than 34 [72]. This reflects the persistent under-vaccination among adult migrants, linked to limited life-course and catch-up immunization provision in Europe [41,72].

Significant gaps remain in understanding access to vaccines (and other medicines [14]) across the entire migration cycle: most studies focused on reception or host countries, while few examined access during departure, transit, or return or deportation phases. These gaps reflect methodological and ethical challenges in researching highly mobile populations. Addressing blind spots in departure, transit, and return phases will require collaborative, cross-border research frameworks and interoperable data systems that can follow people across borders, while assuring data protection through robust mechanisms for deidentification, before data are shared for surveillance and research [73,74]. Legal and administrative precarity clearly undermines universal vaccine access, as shown by delayed immunization of undocumented children or those awaiting their residence permit [52]. These inequities have tangible public health consequences, including outbreaks of measles and hepatitis A in camps and overcrowded settlements [75,76]. The findings mirror previous studies documenting that exclusionary policies, fragmented service provision, and administrative complexity undermine the progress toward universal vaccination coverage in Europe, despite the policy commitment to “health for all” [3,76,77].

We also looked at determinants shaping vaccine uptake among migrants. “Access” was the most frequently reported, followed by acceptance, affordability, and awareness. Activation, i.e., the ability of health systems to effectively reach and engage migrants, remains underexplored. Language barriers, discrimination, and xenophobia limit health-seeking behaviours, while legitimate fears that data may be shared with immigration officers and police, with the risk of deportation or arrest, discourage undocumented migrants from engaging with formal health and vaccination services. At the system level, fragmented data systems and a lack of standardized vaccination records further limited uptake. Overall, there are significant unmet needs in access to vaccination among migrants in or on their way to Europe, with access disparities attributable to various interconnected barriers [14].

### 4.1. Mobility: A Structural Determinant of Access to Vaccines

High population mobility within or across countries contributes to lower coverage for second doses of vaccines, and disrupts follow-up [1,2,3,27,62]. These dynamics illustrate how movement itself hinders access to vaccines, as migrants transition between jurisdictions with different languages, entitlements, health information systems, and vaccination schedules.

Mobility, whether forced or voluntary, plays a central role in shaping exposure to VPDs [8,13]. Most surveillance and research systems assume sedentary populations, yet growing crises driving displacement require health systems to adapt and provide safe, flexible access to vaccines for groups with high mobility. Rather than being framed under concepts of “hard to reach” [78] or “loss to follow-up,” mobility should be recognized as a defining feature of migrant life and the health journey, which requires adaptable and coordinated vaccination approaches. Some humanitarian responses, such as mobile clinics along the Ukrainian borders with Moldova and Poland, offer effective models for delivering vaccinations, especially during transit at border points in conflict-affected areas [79]. This calls for interoperable vaccination records, flexible delivery models and rigorous protection of personal data, ensuring that protection is continuous rather than conditional on stability.

### 4.2. Collecting and Protecting Data

There is a persistent tension between the need for vaccination documentation and follow-up, and the fear of misuse of personal and medical data. Several studies highlighted fragmented vaccination records, reliance on verbal histories, and limited data exchange between providers, undermining the continuity of care [33,35,47,49,63,66,69]. Calls for harmonized immunization registries and cross-border data sharing to ensure access and avoid re-vaccination are common [9,33]. However, there are legitimate fears that the personal and medical data of migrants, especially if undocumented, could be shared with immigration authorities [37,53,64]. Protecting personal data is essential to safeguard individuals’ health and maintain trust in healthcare [3,10]. Data protection is integral to refugee protection, as misuse could expose refugees and asylum seekers to arrest or deportation [80]. Balancing the need for interoperable vaccination systems with strong confidentiality and privacy safeguards is essential to protect the most vulnerable, build trust and promote equitable vaccine access for all.

### 4.3. Policy Implications

Improving vaccine access for migrants in, or on their way to, Europe requires actions beyond service delivery, to address the broader gaps in European vaccination policies. These are generally structured around childhood schedules, thus are poorly aligned with the age profile and life-course needs of migrants. Adult migrants, many of whom have missed recommended vaccines in their countries of origin, are left behind and miss catch-up immunization [41]. Community-centred, culturally adapted approaches and participatory co-design and flexible delivery models, such as outreach and community-based vaccination, are helpful to strengthen vaccine confidence [4,14,34,35,37,41,53,62,64,70]. However, without the alignment of vaccination policy with demographic patterns of migrants and with migration trajectories, major regional vaccine coverage objectives are unlikely to be achieved—including those in the WHO Immunization Agenda 2030. Opportunities to prevent outbreaks and integrate life-saving vaccines will also be missed [2,41].

Importantly, the barriers described in this review do not affect all migrant groups to the same extent. While asylum seekers and refugees often face delays related to registration procedures, mobility, language barriers, and fragmentation of services, they are generally incorporated into formal reception systems and may benefit from specific vaccination programs or health assessments upon arrival [81]. In contrast, undocumented migrants frequently face additional challenges related to exclusion from health coverage, uncertainty regarding entitlement to vaccination, and fear that personal information could be shared with immigration authorities [54]. These concerns may discourage engagement with vaccination services, even where legal entitlements exist. Policies aiming to improve vaccine uptake should therefore move beyond a one-size-fits-all approach and recognize the distinct legal and administrative realities experienced by different migrant groups. For asylum seekers and refugees, strengthening vaccination pathways within reception systems and ensuring continuity of vaccination records across jurisdictions may be particularly important. For undocumented migrants, priorities include guaranteeing access irrespective of legal status, establishing clear firewalls between health and immigration authorities, and providing vaccination through trusted, low-threshold services that minimize fears of detection or deportation.

There is a need to train healthcare workers in culturally sensitive care, and to embed inclusive immunization frameworks in strong data protection frameworks. During the COVID-19 pandemic, some inclusive vaccination campaigns, such as the rapid inclusion of Ukrainian refugees in national vaccination plans, were made possible by political will. However, inclusivity has rarely been extended to other migrant groups [82].

Attention is also needed for the return or deportation phase, especially as some European countries are increasingly calling for the return of certain migrant groups [82]. Free vaccination prior to return is rarely addressed, but is essential for the continuity of health protection of extremely vulnerable persons [4,65]. In the studies included in our sample, Syrians were the most frequently reported nationality, reflecting the fact that they constitute one of the two largest refugee populations in Europe and globally. The growing policy debate on the return or repatriation of Syrian refugees since the fall of the Assad regime heightens the relevance of the return phase of the migration cycle, as a substantial proportion of this group is likely to pass through this pathway [83]. This underscores the importance of strengthening vaccination provision and uptake prior to return to fragile health systems, where immunization programs have been disrupted and access to vaccines for returnees is likely to be constrained.

### 4.4. Strengths and Limitations

Our scoping review provides a comprehensive synthesis of evidence, drawn upon research and grey literature over a 25-year period, on the access to and uptake of vaccines among migrants across different stages of the migration cycle toward and in Europe. We identified structural inequities and systemic barriers, as well as examples of inclusive interventions.

Some limitations should be acknowledged. First, we included only sources in English, which may have led to the exclusion of relevant evidence. Second, although we used multiple databases and snowballing, the absence of EMBASE in our search strategy, motivated by the lack of institutional access at the time of searching, may have limited coverage. Third, some of the included sources were literature reviews that may have contained primary studies also identified independently through our searches. As the purpose of this scoping review was to map and characterize the available evidence rather than to quantitatively synthesize findings, this potential overlap was not expected to affect the interpretation of results, although it may have contributed to greater emphasis on certain themes within the literature. Finally, publication bias cannot be ruled out, as research on migrants’ access to vaccines was more often reported in countries with stronger research capacity or established expertise, or in fields with more availability of funds (e.g., COVID-19), potentially overrepresenting certain settings and priorities.

## 5. Conclusions

Vaccines are inequitably accessed by asylum seekers, refugees, and undocumented migrants on their way to Europe and in Europe, with persistent and systemic access gaps cutting across vaccine types, and migrants’ legal status and age groups. Gaps are particularly pronounced among adults. These inequities highlight a critical missed opportunity to protect individual and population health.

Barriers to vaccine access are shaped by individual-level factors but also by structural, legal, and administrative factors. While community-centred and culturally tailored interventions have shown potential to reduce disparities, their impact will remain limited unless embedded in inclusive national immunization frameworks that guarantee entitlement to vaccination for all migrants, irrespective of legal status, and that ensure unconditional protection of personal and medical data. Sustained progress will depend on political commitment to align national laws with international obligations, to strengthen data systems with robust privacy safeguards, and to foster cross-border coordination to ensure vaccine access, and to maintain it as people move.

Vaccination policies, including newer and risk-based vaccines such as HPV and hepatitis B, must explicitly incorporate migrants of all ages into catch-up and life-course immunization strategies, with particular attention to asylum seekers, undocumented migrants, and people living in camps or detention, or planning to return to their countries of origin. To achieve this shift, health systems will require dedicated funding, targets, and improved recording of migrant-specific vaccination. At the practice level, it is essential to train healthcare workers in life-course vaccination for people with uncertain histories, alongside co-designed and community-led delivery models—such as outreach, out-of-hours services, and vaccination in trusted community settings. Finally, investment in disaggregated data collection and participatory research is needed to identify drivers of under-immunization, address mistrust, and ensure that the needs of highly vulnerable migrant sub-groups are reflected in vaccination strategies.

Future research and policy efforts must recognize mobility as a defining feature of migrant health and move beyond classical sedentary contexts, to design and test vaccination strategies that accompany people on the move, across borders and at stages of displacement, while protecting their confidentiality. This way, research would support and inform health systems toward deploying collaborative and flexible delivery models, including cross-border coordination and outreach services. Achieving equitable vaccine access is essential for both health equity and health security, and it requires treating vaccination as a shared transnational responsibility, grounded in evidence, ethics, public health and human rights.

## Figures and Tables

**Table 1:** Summary of the 5A determinants of vaccine uptake and related barriers.

Barrier/Determinant (5As)	Examples or Specific Issues Reported
Access	Legal barriers; registration requirements; administrative hurdles affecting access to both routine and COVID-19 vaccinations [4,10,26,43,45,53,62]
Affordability	Uncertainty about free provision; transport costs; loss of income due to time away from work [10,37,46,48,54,58]
Awareness	Lack of information about eligibility and vaccination locations [49,51,54,61]
Acceptance	Mistrust; fear of data sharing with immigration authorities; safety concerns; misinformation; cultural/religious beliefs influencing perceived need [34,48,50,54,55,61,64]
Activation	Lack of outreach strategies; limited reminder/recall systems; insufficient opportunistic vaccination; inflexible service models; absence of drop-in or out-of-hours options [10,32,36,39,58].

## Data Availability

Data sharing is not applicable as no datasets were generated and/or analyzed for this study. All data relevant to the study are included in the article or uploaded as online Supplementary Materials.

## Supplementary Materials

The following supporting information can be downloaded at: https://www.mdpi.com/article/10.3390/vaccines14060551/s1, Supplementary File S1: Preferred Reporting Items for Systematic reviews and Meta-Analyses extension for Scoping Reviews (PRISMA-ScR) Checklist; Supplementary File S2: Search terms; Supplementary File S3: Eligibility criteria; Supplementary File S4: Overview of the included studies and grey literature reports (*n* = 47).
