# Supplementary material for: Access to Vaccines Among Asylum Seekers, Refugees, and Undocumented Migrants Across the Migratory Cycle in the European Union, European Economic Area, Switzerland and the United Kingdom: A Scoping Review
Source: Vaccines (Basel). 2026 Jun 22;14(6):551. doi: 10.3390/vaccines14060551 (PMC13307898; doi:10.3390/vaccines14060551)
Supplement: Supplementary file 1 [file vaccines-14-00551-s001.zip › vaccines-4336781-supplementary.pdf]

Supplementary File S1

Preferred Reporting Items for Systematic reviews and Meta-Analyses extension for Scoping Reviews (PRISMA-ScR) Checklist

SECTION	ITEM	PRISMA-ScR CHECKLIST ITEM	REPORTED ON PAGE #
**TITLE**			
Title	1	Identify the report as a scoping review.	1
**ABSTRACT**			
Structured summary	2	Provide a structured summary that includes (as applicable): background, objectives, eligibility criteria, sources of evidence, charting methods, results, and conclusions that relate to the review questions and objectives.	1
**INTRODUCTION**			
Rationale	3	Describe the rationale for the review in the context of what is already known. Explain why the review questions/objectives lend themselves to a scoping review approach.	2
Objectives	4	Provide an explicit statement of the questions and objectives being addressed with reference to their key elements (e.g., population or participants, concepts, and context) or other relevant key elements used to conceptualize the review questions and/or objectives.	2-3
**METHODS**			
Protocol and registration	5	Indicate whether a review protocol exists; state if and where it can be accessed (e.g., a Web address); and if available, provide registration information, including the registration number.	2
Eligibility criteria	6	Specify characteristics of the sources of evidence used as eligibility criteria (e.g., years considered, language, and publication status), and provide a rationale.	3
Information sources*	7	Describe all information sources in the search (e.g., databases with dates of coverage and contact with authors to identify additional sources), as well as the date the most recent search was executed.	3
Search	8	Present the full electronic search strategy for at least 1 database, including any limits used, such that it could be repeated.	3
Selection of sources of evidence†	9	State the process for selecting sources of evidence (i.e., screening and eligibility) included in the scoping review.	3
Data charting process‡	10	Describe the methods of charting data from the included sources of evidence (e.g., calibrated forms or forms that have been tested by the team before their use, and whether data charting was done independently or in duplicate) and any processes for obtaining and confirming data from investigators.	4
Data items	11	List and define all variables for which data were sought and any assumptions and simplifications made.	4-5
Critical appraisal of individual sources of evidence§	12	If done, provide a rationale for conducting a critical appraisal of included sources of evidence; describe the methods used and how this information was used in any data synthesis (if appropriate).	NA
Synthesis of results	13	Describe the methods of handling and summarizing the data that were charted.	4-5

SECTION	ITEM	PRISMA-ScR CHECKLIST ITEM	REPORTED ON PAGE #
**RESULTS**			
Selection of sources of evidence	14	Give numbers of sources of evidence screened, assessed for eligibility, and included in the review, with reasons for exclusions at each stage, ideally using a flow diagram.	5
Characteristics of sources of evidence	15	For each source of evidence, present characteristics for which data were charted and provide the citations.	5-8
Critical appraisal within sources of evidence	16	If done, present data on critical appraisal of included sources of evidence (see item 12).	NA
Results of individual sources of evidence	17	For each included source of evidence, present the relevant data that were charted that relate to the review questions and objectives.	5-8
Synthesis of results	18	Summarize and/or present the charting results as they relate to the review questions and objectives.	5-8
**DISCUSSION**			
Summary of evidence	19	Summarize the main results (including an overview of concepts, themes, and types of evidence available), link to the review questions and objectives, and consider the relevance to key groups.	8
Limitations	20	Discuss the limitations of the scoping review process.	10-11
Conclusions	21	Provide a general interpretation of the results with respect to the review questions and objectives, as well as potential implications and/or next steps.	11
**FUNDING**			
Funding	22	Describe sources of funding for the included sources of evidence, as well as sources of funding for the scoping review. Describe the role of the funders of the scoping review.	11

JBI = Joanna Briggs Institute; PRISMA-ScR = Preferred Reporting Items for Systematic reviews and Meta-Analyses extension for Scoping Reviews.
* Where *sources of evidence* (see second footnote) are compiled from, such as bibliographic databases, social media platforms, and Web sites.
† A more inclusive/heterogeneous term used to account for the different types of evidence or data sources (e.g., quantitative and/or qualitative research, expert opinion, and policy documents) that may be eligible in a scoping review as opposed to only studies. This is not to be confused with *information sources* (see first footnote).
‡ The frameworks by Arksey and O'Malley (6) and Levac and colleagues (7) and the JBI guidance (4, 5) refer to the process of data extraction in a scoping review as data charting.
§ The process of systematically examining research evidence to assess its validity, results, and relevance before using it to inform a decision. This term is used for items 12 and 19 instead of "risk of bias" (which is more applicable to systematic reviews of interventions) to include and acknowledge the various sources of evidence that may be used in a scoping review (e.g., quantitative and/or qualitative research, expert opinion, and policy document).

From: Tricco AC, Lillie E, Zarin W, O'Brien KK, Colquhoun H, Levac D, et al. PRISMA Extension for Scoping Reviews (PRISMAScR): Checklist and Explanation. Ann Intern Med. 2018;169:467–473. doi: 10.7326/M18-0850.

Supplementary file 2: Search terms

PubMed

(("refugees"[MeSH Terms] OR "refugees"[All Fields] OR ("asylum"[All Fields] AND "seeker"[All Fields]) OR "asylum seeker"[All Fields] OR ("refugee s"[All Fields] OR "refugees"[MeSH Terms] OR "refugees"[All Fields] OR "refugee"[All Fields]) OR ("undocumented"[All Fields] AND ("migrant s"[All Fields] OR "transients and migrants"[MeSH Terms] OR ("transients"[All Fields] AND "migrants"[All Fields]) OR "transients and migrants"[All Fields] OR "migrant"[All Fields] OR "migrants"[All Fields])) OR (("illegal"[All Fields] OR "illegality"[All Fields] OR "illegally"[All Fields] OR "illegals"[All Fields]) AND ("migrant s"[All Fields] OR "transients and migrants"[MeSH Terms] OR ("transients"[All Fields] AND "migrants"[All Fields]) OR "transients and migrants"[All Fields] OR "migrant"[All Fields] OR "migrants"[All Fields])) OR (("irregular"[All Fields] OR "irregularities"[All Fields] OR "irregularity"[All Fields] OR "irregulars"[All Fields]) AND ("migrant s"[All Fields] OR "transients and migrants"[MeSH Terms] OR ("transients"[All Fields] AND "migrants"[All Fields]) OR "transients and migrants"[All Fields] OR "migrant"[All Fields] OR "migrants"[All Fields]))) AND ("migrate"[All Fields] OR "migrated"[All Fields] OR "migrates"[All Fields] OR "migrating"[All Fields] OR "migration"[All Fields] OR "migrational"[All Fields] OR "migrations"[All Fields] OR "migrator"[All Fields] OR "migrators"[All Fields] OR ("displace"[All Fields] OR "displaced"[All Fields] OR "displacement, psychological"[MeSH Terms] OR ("displacement"[All Fields] AND "psychological"[All Fields]) OR "psychological displacement"[All Fields] OR "displacement"[All Fields] OR "displacements"[All Fields] OR "displaces"[All Fields] OR "displacing"[All Fields]) OR ("countries"[All Fields] OR "country"[All Fields] OR "country s"[All Fields] OR "countrys"[All Fields]) OR ("geographic locations"[MeSH Terms] OR ("geographic"[All Fields] AND "locations"[All Fields]) OR "geographic locations"[All Fields] OR "region"[All Fields] OR "region s"[All Fields] OR "regional"[All Fields] OR "regionalization"[All Fields] OR "regionalizations"[All Fields] OR "regionalize"[All Fields] OR "regionalized"[All Fields] OR "regionalizing"[All Fields] OR "regionally"[All Fields] OR "regionals"[All Fields] OR "regions"[All Fields]) OR ("transit"[All Fields] OR "transited"[All Fields] OR "transiting"[All Fields] OR "transition"[All Fields] OR "transitional"[All Fields] OR "transitionals"[All Fields] OR "transitioned"[All Fields] OR "transitioning"[All Fields] OR "transitions"[All Fields] OR "transits"[All Fields]) OR ("reception"[All Fields] OR "receptions"[All Fields] OR "receptive"[All Fields]) OR ("host"[All Fields] AND ("countries"[All Fields] OR "country"[All Fields] OR "country s"[All Fields] OR "countrys"[All Fields])) OR (("destination"[All Fields] OR "destinations"[All Fields] OR "destinations"[All Fields] OR "destined"[All Fields]) AND ("countries"[All Fields] OR "country"[All Fields] OR "country s"[All Fields] OR "countrys"[All Fields])) OR ("deport"[All Fields] OR "deportation"[MeSH Terms] OR "deportation"[All Fields] OR "deportations"[All Fields] OR "deported"[All Fields] OR "deporting"[All Fields]) OR ("origin"[All Fields] OR "originate"[All Fields] OR "originated"[All Fields] OR

"originates"[All Fields] OR "originating"[All Fields] OR "origination"[All Fields] OR "originations"[All Fields] OR "origins"[All Fields])) AND ((("access"[All Fields] OR "accessed"[All Fields] OR "accesses"[All Fields] OR "accessibilities"[All Fields] OR "accessibility"[All Fields] OR "accessible"[All Fields] OR "accessing"[All Fields]) AND ("essential"[All Fields] OR "essentials"[All Fields]) AND ("medicin"[All Fields] OR "medicinal"[All Fields] OR "medicinally"[All Fields] OR "medicinals"[All Fields] OR "medicine"[MeSH Terms] OR "medicine"[All Fields] OR "medicine s"[All Fields] OR "medicines"[All Fields])) OR ("continuum"[All Fields] OR "continuums"[All Fields]) OR (("essential"[All Fields] OR "essentials"[All Fields]) AND ("medicin"[All Fields] OR "medicinal"[All Fields] OR "medicinally"[All Fields] OR "medicinals"[All Fields] OR "medicine"[MeSH Terms] OR "medicine"[All Fields] OR "medicine s"[All Fields] OR "medicines"[All Fields])) OR "drug"[All Fields] OR ("vaccin"[Supplementary Concept] OR "vaccin"[All Fields] OR "vaccination"[MeSH Terms] OR "vaccination"[All Fields] OR "vaccinable"[All Fields] OR "vaccinal"[All Fields] OR "vaccinate"[All Fields] OR "vaccinated"[All Fields] OR "vaccinates"[All Fields] OR "vaccinating"[All Fields] OR "vaccinations"[All Fields] OR "vaccination s"[All Fields] OR "vaccinator"[All Fields] OR "vaccinators" OR "vaccine s"[All Fields] OR "vaccined"[All Fields] OR "vaccines"[MeSH Terms] OR "vaccines"[All Fields] OR "vaccine"[All Fields] OR "vaccins"[All Fields]) OR ("medic"[All Fields] OR "medical"[All Fields] OR "medicalization"[MeSH Terms] OR "medicalization"[All Fields] OR "medicalizations"[All Fields] OR "medicalize"[All Fields] OR "medicalized"[All Fields] OR "medicalizes"[All Fields] OR "medicalizing"[All Fields] OR "medically"[All Fields] OR "medicals"[All Fields] OR "medicated"[All Fields] OR "medication s"[All Fields] OR "medics"[All Fields] OR "pharmaceutical preparations"[MeSH Terms] OR ("pharmaceutical"[All Fields] AND "preparations"[All Fields]) OR "pharmaceutical preparations"[All Fields] OR "medication"[All Fields] OR "medications"[All Fields]) OR ("biopharmaceutics"[MeSH Terms] OR "biopharmaceutics"[All Fields] OR "pharmaceutic"[All Fields] OR "pharmaceutics"[All Fields] OR "pharmaceutical preparations"[MeSH Terms] OR ("pharmaceutical"[All Fields] AND "preparations"[All Fields]) OR "pharmaceutical preparations"[All Fields] OR "pharmaceutical"[All Fields] OR "pharmaceuticals"[All Fields] OR "pharmaceutical s"[All Fields] OR "pharmaceutically"[All Fields]) OR ("availabilities"[All Fields] OR "availability"[All Fields] OR "available"[All Fields]) OR ("afford"[All Fields] OR "affordable"[All Fields] OR "affordably"[All Fields] OR "afforded"[All Fields] OR "affording"[All Fields] OR "affords"[All Fields] OR "costs and cost analysis"[MeSH Terms] OR ("costs"[All Fields] AND "cost"[All Fields] AND "analysis"[All Fields]) OR "costs and cost analysis"[All Fields] OR "affordability"[All Fields]) OR (("barrier"[All Fields] OR "barrier s"[All Fields] OR "barriers"[All Fields]) AND ("access"[All Fields] OR "accessed"[All Fields] OR "accesses"[All Fields] OR "accessibilities"[All Fields] OR "accessibility"[All Fields] OR "accessible"[All Fields] OR "accessing"[All Fields]) AND ("medicin"[All Fields] OR "medicinal"[All Fields] OR "medicinally"[All Fields] OR "medicinals"[All Fields] OR "medicine"[MeSH Terms] OR "medicine"[All Fields] OR

"medicine s"[All Fields] OR "medicines"[All Fields])) OR (("biopharmaceutics"[MeSH Terms] OR "biopharmaceutics"[All Fields] OR "pharmaceutic"[All Fields] OR "pharmaceutics"[All Fields] OR "pharmaceutical preparations"[MeSH Terms] OR ("pharmaceutical"[All Fields] AND "preparations"[All Fields]) OR "pharmaceutical preparations"[All Fields] OR "pharmaceutical"[All Fields] OR "pharmaceuticals"[All Fields] OR "pharmaceutical s"[All Fields] OR "pharmaceutically"[All Fields]) AND ("drug delivery systems"[MeSH Terms] OR ("drug"[All Fields] AND "delivery"[All Fields] AND "systems"[All Fields]) OR "drug delivery systems"[All Fields] OR "system"[All Fields] OR "system s"[All Fields] OR "systems"[All Fields])))) AND ((english[Filter]) AND (2000:2022[pdat]))

<u>CINHAL</u>

Search String- (ALL ("asylum" AND "seeker" OR "refugees" OR "undocumented" AND "migrants" OR "illegal" OR "illegally" OR "illegality" OR "illegals" AND "migrants" OR "transients" AND "migrants" OR "irregular" OR "irregularities" OR "irregularity" OR "irregulars" AND "migrants") AND ALL ("migrate" OR "migrated" OR "migrates" OR "migrating" OR "migration" OR "migrational" OR "migrations" OR "migrator" OR "migrators" OR "displace" OR "displaced" OR "displacement" OR "destined" OR "countries" OR "country" OR "country s" OR "geographic locations" OR "geographic" AND "locations" OR "region s" OR "regional" OR "region" OR "regionals" OR "regions" AND "origins" OR "transit" OR "transited" OR "transiting" OR "transition" OR "transitional" OR "transitionals" OR "transitioned" OR "transitioning" OR "transitions" OR "transits" OR "reception" OR "receptions" OR "receptive" OR "host" AND "countries" OR "country" OR "countries" AND "destinations" OR "deport" OR "deportation" OR "deportations" OR "deported" OR "deporting" OR "origin" OR "originate" OR "originated" OR "originates" OR "originating" OR "origination" OR "originations" OR "origins") AND ALL ("accessed" OR "accesses" OR "accessibilities" OR "accessibility" OR "accessible" OR "accessing" OR "access" AND "essential" OR "essentials" AND "medicines" OR "medicinal" OR "medicinally" OR "medicinals" OR "medication" OR "medicalization" OR "drug" OR "vaccine" OR "vaccinations" OR "vaccination s" OR "pharmaceuticals" OR "availabilities" OR "availability" OR "available" OR "affordability" OR "afforded" OR "affording" OR "affords" OR "barrier" OR "barriers" OR "barrier s" AND "access" AND "medicine" OR "pharmaceuticals" OR "pharmaceutical" AND "system "OR "system s" OR "systems"))

Limiters - Published Date: 20000101-20220931; English Language; Peer Reviewed; Research Article; Exclude MEDLINE records; Evidence-Based Practice; Human; Geographic Subset: Europe; Language: English

Expanders - Apply related words; Also search within the full text of the articles; Apply equivalent subjects

Search modes - Find all my search terms

Scopus

(ALL ("asylum" AND "seeker" OR "refugees" OR "undocumented" AND "migrants" OR "illegal" OR "illegally" OR "illegality" OR "illegals" AND "migrants" OR "transients" AND "migrants" OR "irregular" OR "irregularities" OR "irregularity" OR "irregulars" AND "migrants") AND ALL ("migrate" OR "migrated" OR "migrates" OR "migrating" OR "migration" OR "migrational" OR "migrations" OR "migrator" OR "migrators" OR "displace" OR "displaced" OR "displacement" OR "countries" OR "country" OR "country s" OR "geographic locations" OR "geographic" AND "locations" OR "region s" OR "regional" OR "region" OR "regionals" OR "regions" AND "origins" OR "transit" OR "transited" OR "transiting" OR "transition" OR "transitional" OR "transitionals" OR "transitioned" OR "transitioning" OR "transitions" OR "transits" OR "reception" OR "receptions" OR "receptive" OR "host" AND "countries" OR "country" OR "countries" AND "destinations" "destined" OR "deport" OR "deportation" OR "deportations" OR "deported" OR "deporting" OR "origin" OR "originate" OR "originated" OR "originates" OR "originating" OR "origination" OR "originations" OR "origins") AND ALL ("accessed" OR "accesses" OR "accessibilities" OR "accessibility" OR "accessible" OR "accessing" OR "access" AND "essential" OR "essentials" AND "medicines" OR "medicinal" OR "medicinally" OR "medicinals" OR "medication" OR "medicalization" OR "drug" OR "vaccine" OR "vaccinations" OR "vaccination s" OR "pharmaceuticals" OR "availabilities" OR "availability" OR "available" OR "affordability" OR "afforded" OR "affording" OR "affords" OR "barrier" OR "barriers" OR "barrier s" AND "access" AND "medicine" OR "pharmaceuticals" OR "pharmaceutical" AND "system "OR "system s" OR "systems")) AND PUBYEAR > 1999 AND PUBYEAR < 2023 AND (LIMIT-TO (DOCTYPE , "ar") OR LIMIT-TO (DOCTYPE , "re") OR LIMIT-TO (DOCTYPE , "ed")) AND (LIMIT-TO (LANGUAGE , "English")) AND (LIMITTO (SRCTYPE , "j"))

Web of Science

(ALL= ("asylum" AND "seeker" OR "refugees" OR "undocumented" AND "migrants" OR "illegal" OR "illegally" OR "illegality" OR "illegals" AND "migrants" OR "transients" AND "migrants" OR "irregular" OR "irregularities" OR "irregularity" OR "irregulars" AND "migrants") AND ALL ("migrate" OR "migrated" OR "migrates" OR "migrating" OR "migration" OR "migrational" OR "migrations" OR "migrator" OR "migrators" OR "displace" OR "displaced" OR "displacement" OR "countries" OR "country" OR "country s" OR "geographic locations" OR "geographic" AND "locations" OR "region s" OR "regional" OR "region" OR "regionals" OR "regions" AND "origins" OR "transit" OR "transited" OR "transiting" OR "transition" OR "transitional" OR "transitionals" OR "transitioned" OR "transitioning" OR "transitions" OR "transits" OR "reception" OR "receptions" OR "receptive" OR "host" AND "countries" OR "country" OR "countries" AND "destinations" OR "deport" OR "deportation" OR "deportations" OR "deported" OR

"deporting" OR "destined" OR "origin" OR "originate" OR "originated" OR "originates" OR "originating" OR "origination" OR "originations" OR "origins") AND ALL ("accessed" OR "accesses" OR "accessibilities" OR "accessibility" OR "accessible" OR "accessing" OR "access" AND "essential" OR "essentials" AND "medicines" OR "medicinal" OR "medicinally" OR "medicinals" OR "medication" OR "medicalization" OR "drug" OR "vaccine" OR "vaccinations" OR "vaccination s" OR "pharmaceuticals" OR "availabilities" OR "availability" OR "available" OR "affordability" OR "afforded" OR "affording" OR "affords" OR "barrier" OR "barrier s" OR "barriers" AND "access" AND "medicine" OR "pharmaceuticals" OR "pharmaceutical" AND "system "OR "system s" OR "systems")) AND 2000 or 2001 or 2002 or 2003 or 2004 or 2005 or 2006 or 2007 or 2008 or 2009 or 2010 or 2011 or 2022 or 2021 or 2020 or 2019 or 2018 or 2017 or 2016 or 2015 or 2014 or 2013 or 2012 (Publication Years) AND English (Languages)

Cochrane Database of Systematic Reviews

("asylum" AND "seeker" OR "refugees" OR "undocumented" AND "migrants" OR "illegal" OR "illegally" OR "illegality" OR "illegals" AND "migrants" OR "transients" AND "migrants" OR "irregular" OR "irregularities" OR "irregularity" OR "irregulars" AND "migrants”: ti,ab,kw AND "migrate" OR "migrated" OR "migrates" OR "migrating" OR "migration" OR "migrational" OR "migrations" OR "migrator" OR "migrators" OR "displace" OR "displaced" OR "displacement" OR "countries" OR "country" OR "country s" OR "geographic locations" OR "geographic" AND "locations" OR "region s" OR "regional" OR "region" OR "regionals" OR "regions" AND "origins" OR "transit" OR "transited" OR "transiting" OR "transition" OR "transitional" OR "transitionals" OR "transitioned" OR "transitioning" OR "transitions" OR "transits" OR "reception" OR "receptions" OR "receptive" OR "host" AND "countries" OR "country" OR "countries" AND "destinations" OR "destined" OR "deport" OR "deportation" OR "deportations" OR "deported" OR "deporting" OR "origin" OR "originate" OR "originated" OR "originates" OR "originating" OR "origination" OR "originations" OR "origins":ti,ab,kw AND "accessed" OR "accesses" OR "accessibilities" OR "accessibility" OR "accessible" OR "accessing" OR "access" AND "essential" OR "essentials" AND "medicines" OR "medicinal" OR "medicinally" OR "medicinals" OR "medication" OR "medicalization" OR "drug" OR "vaccine" OR "vaccinations" OR "vaccination s" OR "pharmaceuticals" OR "availabilities" OR "availability" OR "available" OR "affordability" OR "afforded" OR "affording" OR "affords" OR "barrier" OR "barriers" OR "barrier s" AND "access" AND "medicine" OR "pharmaceuticals" OR "pharmaceutical" AND "system "OR "system s" OR "systems"):ti,ab,kw with Publication Year from 2000 to 2022, with Cochrane Library publication date Between Jan 2000 and Sep 2022, in Trials (Word variations have been searched

Supplementary file S3: Eligibility criteria

Domain	Eligibility criteria	
	Inclusion Criteria	Exclusion criteria
**Time restriction**	Literature (including grey literature) published between 01.01.2000-31.12.2024	Literature published before 01.01.2000
**Language restriction**	Literature available in English	Literature published in other languages
**Population**	Asylum seeker, refugees, undocumented migrants.	Regular migrants, international students, migrants in general without any reference to the legal status of this migrant population that can determine whether this population includes asylum seekers, refugees, or undocumented migrants, or not.
**Intervention**	Access to essential medicines and vaccines; barriers to access to essential medicines and vaccines	Access to healthcare in general, without any reference to access to medicines.
**Setting**	Country of origin, transit, destination (EU/EEA, Switzerland and the UK), deportation settings.	No-EU countries of destination
**Study type**	Qualitative, quantitative, mixed methods studies, reviews and meta-analysis, grey literature, UN agency reports, NGOs reports.	None

Supplementary file S4: Overview of the included studies and grey literature reports (n=47)

Author	Title	Year of publication	Reported vaccines	Country/ies or region	Study method classification	Migrants included	Type of publication
Bentivegna et al	Access to COVID-19 Vaccination during the Pandemic in the Informal Settlements of Rome.	2022	Covid-19	Italy	Qualitative and quantitative.	Asylum seekers, refugees and undocumented migrants	Research study
Elharake et al.	Country immunization policies for refugees across 20 low-middle income and 20 high-income countries.	2022	Multiple vaccines	20 LMICs and 20 HICs	Quantitative	Refugees	Research study
Malchrzak et al.	COVID-19 Vaccination and Ukrainian Refugees in Poland during Russian–Ukrainian War—Narrative Review	2022	Covid-19	Poland	Review	Refugees	Research study
Carter et al.	"We don't routinely check vaccination background in adults": a national qualitative study of barriers and facilitators to vaccine delivery and uptake in adult migrants through UK primary care	2022	Multiple vaccines	UK	Qualitative	Asylum seekers, refugees and undocumented migrants	Research study

Crawshaw et al.	Defining the determinants of vaccine uptake and undervaccination in migrant populations in Europe to improve routine and COVID-19 vaccine uptake: a systematic review	2022	Covid-19	e UK, Switzerland, or one of 30 EU or EEA countries	Review	Asylum seekers, refugees and undocumented migrants	Research study
Hargreaves et al.	Divergent approaches in the vaccination of recently arrived migrants to Europe: a survey of national experts from 32 countries, 2017	2017	Multiple vaccines	EU/EEA countries and Switzerland	Quantitative	Asylum seekers	Research study
Godoy-Ramirez et al.	Exploring childhood immunization among undocumented migrants in Sweden - following qualitative study and the World Health Organizations Guide to Tailoring Immunization Programmes (TIP).	2019	Multiple vaccines	Sweden.	Qualitative	Undocumented migrants	Research study
Møller	Human papillomavirus immunization uptake among girls with a refugee background compared with Danish-born girls: a national register-based cohort study.	2018	HPV	Denmark	Quantitative	Refugees	Research study
Giambi et al.	Immunisation of migrants in EU/EEA countries: Policies and practices	2019	Multiple vaccines	29 EU/EEA countries	Quantitative	Asylum seekers, refugees and undocumented migrants	Research study

Dalla Zuanna et al.	Immunization Offer Targeting Migrants: Policies and Practices in Italy	2018	Multiple vaccines	Italy	Quantitative	Refugees and unaccompanied migrants	Research study
Mellou et al.	Increasing childhood vaccination coverage of the refugee and migrant population in Greece through the European programme PHILOS, April 2017 to April 2018	2019	Multiple vaccines	Greece	Quantitative	Refugees and asylum seekers	Research study
Perry et al.	Inequalities in vaccination coverage and differences in follow-up procedures for asylum-seeking children arriving in Wales, UK	2019	Multiple vaccines	UK	Quantitative	Asylum seekers	Research study
Deal et al.	Immunisation status of UK-bound refugees between January, 2018, and October, 2019: a retrospective, population-based cross-sectional study	2022	Multiple vaccines	UK	Quantitaive	Refugees	Research study
Ravensbergen et al.	National approaches to the vaccination of recently arrived migrants in Europe: A comparative policy analysis across 32 European countries	2019	Multiple vaccines	EU/EEA countries and Switzerland	Qualitative interviews and systematic search in the literature	Migrants (including refugees)	Research study

Giambi et al.	National immunization strategies targeting migrants in six European countries	2019	Multiple vaccines	Croatia, Greece, Italy, Malta, Portugal and Slovenia	Quantitative	NA	Research study
Nichol et al.	Rapid Review of COVID-19 Vaccination Access and Acceptance for Global Refugee, Asylum Seeker and Undocumented Migrant Populations	2022	Covid-19	Countries hosting refugees, asylum seekers, and undocumented migrants (they did not mention the list of the countries).	Review	Refugees, asylum seekers, and undocumented migrants	Research study
Lambert et al.	Reducing burden from respiratory infections in refugees and immigrants: a systematic review of interventions in OECD EU EEA and EU-applicant countries.	2021	Multiple vaccines	OECD, EU, EEA and EU-applicant countries	Review	Refugees and asylum seekers	Research study
Deal et al.	Strategies and action points to ensure equitable uptake of COVID-19 vaccinations: A national qualitative interview study to explore the views of undocumented migrants, asylum seekers, and refugees	2021	Covid-19	UK	Qualitative	Refugees and undocumented migrants	Research study
Matlin at al.	The Challenge of Reaching Undocumented Migrants with COVID-19 Vaccination	2022	Covid-19	EU countries	Qualitative	Asylum seekers, refugees and undocumented migrants	Research study

Hui et al	Interventions to Improve Vaccination Uptake and Cost Effectiveness of Vaccination Strategies in Newly Arrived Migrants in the EU/EEA: A Systematic Review	2018	Multiple vaccines	EU/EEA countries	Review	Asylum seekers, refugees and undocumented migrants	Research study
Vita et al.	Vaccination campaign strategies in recently arrived migrants: experience of an Italian reception centre	2019	Multiple vaccines	Italy	Quantitative	Asylum seekers	Research study
Prymula et al.	Vaccination in newly arrived immigrants to the European Union	2018	Multiple vaccines	EU countries	Review	Refugees and undocumented migrants	Research study
Parellada et al.	Vaccination needs and use of preventive healthcare services among undocumented migrant children in Denmark	2021	Multiple vaccines	Denmark	Review	Undocumented migrants	Research study
Mipatrini et al.	Vaccinations in migrants and refugees: a challenge for European health systems. A systematic review of current scientific evidence	2017	Multiple vaccines	Europe	Review	Asylum seekers and refugees	Research study

Crawshaw et al.	What must be done to tackle vaccine hesitancy and barriers to COVID-19 vaccination in migrants?	2021	Covid-19	NA	NA	Asylum seekers, refugees, and undocumeneted migrants	Research study
Ekezie et al	Access to Vaccination among Disadvantaged, Isolated and Difficult-to-Reach Communities in the WHO European Region: A Systematic Review	2022	Multiple vaccines	WHO European countries	Review	Refugees and asylum seekers	Research study
Farmakioti et al	Access to Healthcare Services and Essential Medicines in GREEK Migrant Camps: an Online Cross-Sectional Study	2023	Multiple vaccines	Greece	Quantitaitve	Refugees and asylum seekers	Research study
Rzymski et al	Vaccination of Ukrainian Refugees: Need for Urgent Action	2022	Multiple vaccines	Poland	Review	Refugees	Research study
Page et al	COVID-19 vaccine hesitancy among undocumented migrants during the early phase of the vaccination campaign: a multicentric cross-sectional study	2022	Covid-19	France, Switzerland and Italy	Quantitative	Undocumented migrants	Research study

Jaeger et al	Paediatric refugees from Ukraine: guidance for health care providers	2022	Multiple vaccines	Switzerland	Qualitative	Refugees	Research study
Cortier et al	Health status and healthcare trajectory of vulnerable asylum seekers hosted in a French Reception Center	2022	Multiple vaccines	France	Quantitaitve	Asylum seekers	Research study
Turatto et al	Ensuring Equitable Access to the COVID-19 Vaccine: The Experience of A Local Health Unit in Rome, Italy	2022	Covid-19	Italy	Qualitative	Undocumented migrants	Research study
Knights et al	Strengthening life-course immunisation in migrant populations: access, equity, and inclusion	2024	Multiple vaccines	European	NA	Migrants (Including refugees)	Research study
Deal et al.	Defining drivers of under-immunization and vaccine hesitancy in refugee and migrant populations	2023	Multiple vaccines	Global (Incl EU Countries)	Review	Refugees, asylum-seekers and migrants	Research study

Burns et al	COVID-19 vaccination coverage for half a million non-EU migrants and refugees in England	2023	Covid-19	England	Quantitaive	Refugees	Research study
Deal et al.	Understanding the views of adult migrants around catch-up vaccination for missed routine immunisations to define strategies to improve coverage: A UK in-depth interview study	2024	Multiple vaccines	UK	Qualitative	Migrants (Including refugees)	Research study
Puchner et al	COVID-19 vaccination roll-out and uptake among refugees and migrants in Greece: a retrospective analysis of national vaccination routine data	2024	Covid-19	Greece	Quantitative	Refugees	Research study
Crawshaw et al.	Navigating vaccination choices: The intersecting dynamics of institutional trust, belonging and message perception among Congolese migrants in London, UK (a reflexive thematic analysis)	2024	Covid-19	UK	Qualitative	Migrants (Including refugees)	Research study
Scarso et al	Access to Vaccination for Newly Arrived Migrants: Developing a General Conceptual Framework	2023	Multiple vaccines	Germany, Poland, Spain, Italy, Greece, Malta, and Cyprus,	Qualitative	Migrants (including asylum seekers)	Research study

WHO	Provision of immunization services to refugees: guidance for host countries in the context of mass population movement from Ukraine.	2022	Multiple vaccines	Europe	Operational report	Refugees	Grey literature report
WHO	Access to vaccination for refugees from Ukraine needed to protect the most vulnerable among them from vaccine-preventable diseases.	2022	Multiple vaccines	Ukraine	Operational report	Refugees	Grey literature report
IOM	Ensuring Migrants' Equitable Access to COVID-19 Vaccines	2021	Covid-19	NA	Operational report	Refugees, asylum seekers, migrants	Grey literature report
WHO	World Health Organization COVID-19 Immunization in Refugees and Migrants: Principles and Key Considerations.	2021	Covid-19	NA	Operational report	Refugees	Grey literature report
WHO	Delivery of immunization services for refugees and migrants: technical guidance	2019	Multiple vaccines	Europe	Review	Refugees	Grey literature report

WHO	Joint Statement on general principles on vaccination of refugees, asylum-seekers and migrants in the WHO European Region	2015	Multiple vaccines	Europe	Operational report	Refugees, asylum-seekers and migrants	Grey literature report
European Centre For Disease Prevention and Control	Public health guidance on screening and vaccination for infectious diseases in newly arrived migrants within the EU/EEA	2018	Multiple vaccines	EU/EEA	Operational report	Refugees	Grey literature report
UNHCR	UNHCR - Public Health 2019, Annual Global Overview	2019	Multiple vaccines	NA	Operational report	Refugees	Grey literature report
